# Impact of Structural Stiffness on Vibration Periods of Concrete Buildings: A Systematic Review

**DOI:** 10.3390/ma18112612

**Published:** 2025-06-03

**Authors:** Juan Paredes, Wladimir Ramirez, Fernanda Pico, Rodrigo Acosta, Oscar G. Toapanta, Margarita Mayacela

**Affiliations:** 1 Facultad de Ingeniería Civil y Mecánica, Universidad Técnica de Ambato, Ambato 180104, Ecuador; wj.ramirez@uta.edu.ec (W.R.); mf.pico@uta.edu.ec (F.P.); ri.acosta@uta.edu.ec (R.A.); cm.mayacela@uta.edu.ec (M.M.); 2 GI3M—Grupo de Investigación e Innovación en Ingeniería Mecánica, Universidad Técnica de Ambato, Ambato 180104, Ecuador; 3 GeReNIS—Grupo de Investigación Gestión de Recursos Naturales e Infraestructura Sustentable, Universidad Técnica de Ambato, Ambato 180104, Ecuador; 4 Campus Benjamín Araujo, Instituto Superior Tecnológico Pelileo, Patate 180550, Ecuador; oscartoapantaambjlm@gmail.com

**Keywords:** stiffness, vibration periods, concrete buildings, structural models, dynamic response

## Abstract

Research on the relationship between structural characteristics and vibration periods in concrete buildings is crucial to ensure the safety and efficiency of these structures, especially in earthquake-prone areas. This article aims to analyze and compare the impact of structural stiffness of different elements, such as beams, columns and shear walls, on vibration periods, through a systematic review of existing models and formulas in the literature, identifying their applications and limitations. The methodology used consists of a systematic review that integrates recent and relevant studies, providing a solid basis for analysis. The results indicate that an increase in the stiffness of structural elements can reduce vibration periods by 20–50%, implying a faster response to external forces. Even in low-rise buildings, the fundamental period can be reduced by 53% to 70%. These findings are significant for the design and construction of concrete buildings, as they suggest that incorporating rigid structural elements can improve seismic resistance and reduce the risk of damage. In addition, the research contributes to the field of structural dynamics by highlighting the need to integrate different methods of analysis and evaluation. It is recommended that engineers and architects adopt innovative approaches, such as the use of emerging technologies and monitoring methods, to improve damage detection and optimize structural design. Finally, it identifies areas where more research is required, suggesting that future studies should explore the interaction between structural characteristics and environmental conditions for a more complete understanding of the vibrational behavior of buildings.

## 1. Introduction

The relationship between structural characteristics, such as stiffness and height, and vibration periods of concrete buildings is a fundamental area of study in structural engineering; This relationship is essential to understand how buildings respond to dynamic loads, such as those from earthquakes, and to ensure their safety and performance. The fundamental period of vibration is influenced by a variety of factors, such as building height, stiffness, and material properties, which are crucial for accurate seismic design and analysis [1,2]. The stiffness of a structure directly influences its dynamic behavior, affecting its ability to withstand seismic loads and environmental vibrations [3,4,5]. Understanding this relationship is critical to designing buildings that are not only safe, but also cost- and resource-efficient [6,7].

**Figure 1:**
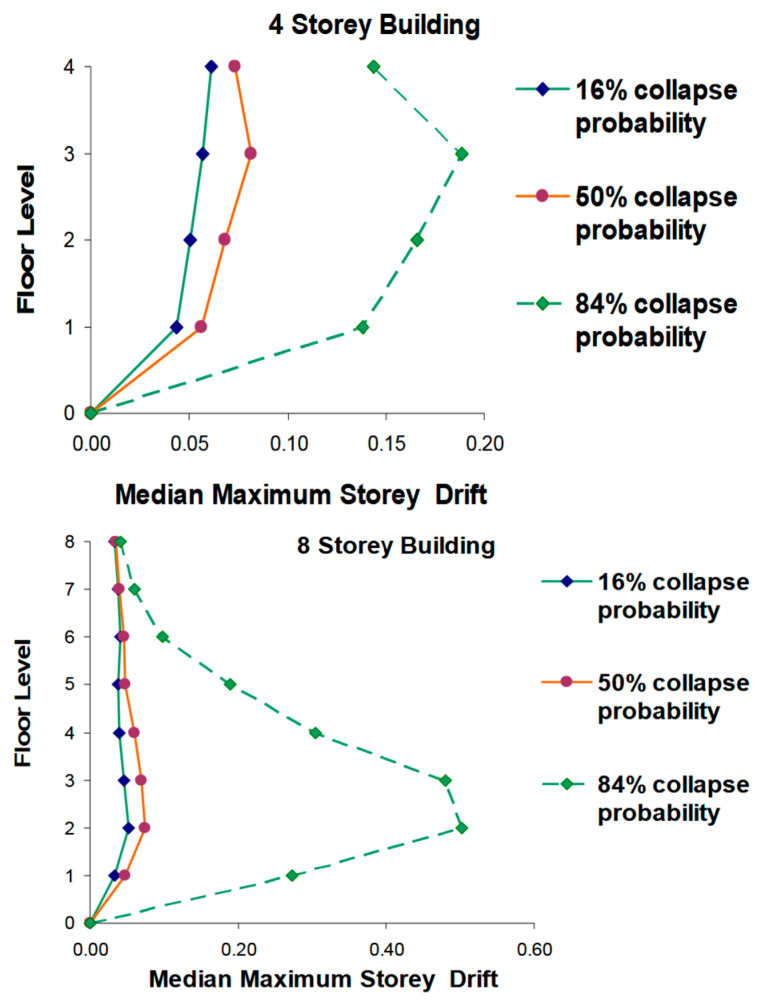
Median maximum story drifts over IDA results (4- and 8-story buildings) [15].

The vibration periods of a structure are determined by its mass and stiffness, where stiffness refers to the resistance of the structure to deform under load [8,9]. At the same time, it is considered that the modification of stiffness, height and cracking influences the calculation of the fundamental period of vibration in buildings of different structural systems. The results show that an increase in the stiffness of the building reduces the vibration period, implying a faster response to external forces. In addition, it is observed that height and cracking also significantly affect the fundamental period of vibration [10,11]. In this context, stiffness can be influenced by various structural elements, including shear walls, columns, and infill or infill panels [9,12,13]. Recent literature has explored how different configurations of these elements affect global stiffness and, therefore, periods of vibration [3,14]. Figure 1 shows the investigation of the maximum mezzanine drift in 4- and 8-story buildings under incremental dynamic analysis (IDA), considering collapse probabilities of 16%, 50% and 84%. It is observed that the tallest buildings present greater deformations on the upper levels, while in the lower ones the deformations are concentrated on the intermediate floors [15]. This highlights the influence of structural stiffness and mass distribution on seismic response, being key to optimizing design and improving structural safety.

Recent research has highlighted the critical role that both height and stiffness play in assessing the vibration periods of concrete buildings, using a combination of experimental and numerical methods [16]. These studies underscore the importance of accurately predicting the fundamental period of vibration, which is crucial for seismic design and structural integrity assessment. The need to take into account various factors, such as filler elements, material properties and damage states, is underlined to improve the accuracy of these predictions [4,14]. In Domenico’s research, it is explained that numerical analyses, such as incremental time-history analyses, help establish relationships between damage states and period lengthening, which can be used to assess structural usability after seismic events [17].

While these studies provide valuable insights into the factors that affect vibration periods, it is also important to consider the limitations of current empirical formulas and the need to continually update seismic design codes. The dynamic properties of buildings can vary significantly depending on the local effects of the site and the characteristics of the materials, so further research and refinement of prediction models is needed [18,19]. Likewise, studies have shown that the relationship between the height of a building and its vibration period can be non-linear, depending on the stiffness of the structural elements [10,12]. In addition, the influence of soil conditions and environmental characteristics has also been studied, revealing that these variables can significantly alter vibration periods [3,9].

Numerous studies have addressed the relationship between stiffness and vibration periods in concrete buildings. For example, Ditommaso [6] He analyzed how the stiffness of infill panels affects the vibration periods in reinforced concrete structures, finding that the inclusion of these elements can significantly reduce the fundamental period. Likewise, Kaplan [20] He investigated the relationship between height and vibration period in mid-rise buildings, providing empirical formulas that have been widely adopted in design practice. Other studies, such as that of Ornthammarath [21], They have used neural networks to estimate fundamental periods, highlighting the importance of stiffness in their models. The latest advances use machine learning to predict vibration periods more accurately, incorporating variables such as the number of plants and opening rates [22]. Other authors propose novel alternatives with the use of infill panels that can reduce the fundamental period by 53% to 70% in low-rise buildings [23], and the arrangement of the infill walls affects the lateral load-bearing capacity and overall structural performance [24,25].

Despite these advances, gaps persist in the literature that justify the need for a systematic review. Many studies focus on specific cases or a single type of structural element, which limits the generalizability of the results [6,26]. Studies highlight the importance of an integrated approach that simultaneously considers factors such as building height, structural stiffness, and soil conditions in the analysis of vibration periods. There are elements that affect exposure to transport-induced vibrations in buildings, suggesting that a holistic approach is essential to properly assess vibration transmission [27]. The use of empirical formulas and artificial neural networks to estimate vibration periods is proposed, pointing out that the accuracy of these estimates depends on properly integrating the three variables [7]. In addition, to correctly assess seismic responses and vibration suitability in hybrid buildings, it is crucial to consider these factors together, thus improving the accuracy of the analyses [28,29].

The literature review reveals several significant gaps in the current research, as there are critical gaps in the understanding of how structural stiffness and soil conditions affect vibration periods in concrete buildings. We can start by reviewing that there is a scarcity of studies that comparatively analyze the impact of different elements of stiffness on periods of vibration [30]. There is a notable lack of studies that systematically compare the effects of various elements of stiffness on periods of vibration [31]. Research indicates that different levels of structural stiffness can significantly influence vibration responses, as demonstrated in soil-structure interaction (SSI) studies [32]. Also, most existing models do not adequately consider variations in soil conditions and their influence on structural stiffness [7,33]. These existing models often overlook the variability of soil conditions, which can drastically alter structural stiffness and vibration behavior [34]. For example, studies on subway vibrations reveal that geological conditions, such as soft soil versus rock strata, affect vibration transmission characteristics [35].

Finally, the absence of an integrated theoretical framework limits the ability of engineers to effectively apply discoveries in design contexts [2]. A solid framework could facilitate better predictions of vibration behavior, leading to safer and more efficient building designs. Conversely, while it is crucial to address these gaps, some researchers argue that focusing solely on stiffness and soil conditions may overlook other important factors, such as dynamic load and environmental influences, which also play a vital role in vibration dynamics.

The objective of this article is to analyze and compare the impact of the structural stiffness of different elements on the vibration periods of concrete buildings, through a systematic review of existing models and formulas in the literature, identifying their applications and limitations. This objective seeks to fill the identified thematic gaps and advance knowledge of the field, providing a solid foundation for future research and practical applications in the design of concrete structures.

## 2. Methods

This systematic review was conducted in accordance with the PRISMA 2020 reporting guidelines. The review was not registered in a prospective registry such as PROSPERO or INPLASY due to the non-medical scope of the topic. All methodological steps, including the search strategy, inclusion/exclusion criteria, data extraction, and synthesis, are described in detail to ensure transparency and reproducibility.

Comprehensive literature searches were performed in major scientific databases, including Scopus, Web of Science, and ScienceDirect. The search covered all publication years available up to the end of 2023 and included manual screening of reference lists to ensure a broad inclusion of relevant studies. Duplicate records were removed using Mendeley Reference Manager software. A total of 480 records were initially identified, 360 screened by title and abstract, and 100 full-text articles were assessed for eligibility. The final synthesis included 53 studies that met all predefined inclusion criteria

In this section, a systematic review of existing models and formulas in the literature that address the relationship between structural characteristics and vibration periods of concrete buildings will be conducted. The focus will be on analyzing and comparing the impact of the structural stiffness of different elements on vibration periods. This review will be structured into several components and methodological approaches, which are described below.

### 2.1. Mathematical and Theoretical Models

The main formulas and mathematical models used to calculate vibration periods in concrete buildings will be reviewed, emphasizing how the stiffness of various elements (beams, columns, shear walls) influences these periods. Several empirical and theoretical models have been developed to relate structural stiffness—derived from elements such as beams, columns, and shear walls—to the fundamental period of vibration. This synthesis highlights the main methodologies and conclusions of the recent literature as empirical models often relate the fundamental period of vibration to the height of the building, with studies suggesting that taller buildings exhibit longer periods [2]. Research has shown that fill elements can significantly improve lateral stiffness, affecting the natural period and spectral acceleration during seismic events [14,36].

Incorporating effective stiffness into beams, columns, and walls can lead to a noticeable increase in vibration periods, with findings indicating a 1.2-fold increase in the period when gross inertia is reduced [37]. Effective stiffness takes into account factors such as axial loads and cracked regions, which are critical to making accurate predictions of seismic response [37]. In addition, a method for the analysis of vibrations of asymmetric buildings using the transfer matrix technique was proposed. The transfer matrix technique has been proposed to analyze asymmetrical buildings, treating them as beams equivalent to shear and torsion, allowing a more nuanced understanding of vibratory behavior [37,38].

Dynamic testing of high-rise buildings has led to the development of empirical adjustment formulas that consider structural types and conditions of use, improving the accuracy of estimates of vibration periods [18]. While these models provide valuable information, it is essential to recognize that variations in building design and local seismic conditions can result in discrepancies between predicted and actual vibration periods. This highlights the need for continuous refinement of empirical formulas and analytical methods in structural engineering.

The most used models include the finite element method, which allows for a detailed representation of material geometry and properties, and the modal analysis method, which breaks down the dynamic response of the structure into its natural modes [3,9]. The FEM was employed to analyze the dynamic properties of this concrete gravity dam, revealing a fundamental natural frequency of 0.0039 mm at the ridge, underscoring the importance of controlling modal shapes [39]. FEM and modal analysis were used to assess the dynamic characteristics of UHPC beams, correlating structural stiffness and natural frequency, which is vital for structural health monitoring [1].

Extensive modal analyses of 382 building models revealed the impact of design parameters such as column height and elasticity on the fundamental vibration period, leading to the development of a predictive model [2]. Studies that have applied these methods to calculate the vibration periods in different types of concrete structures will be explored, highlighting the variations in the results according to the stiffness of the structural elements considered [40]. Although FEM and modal analysis provide detailed information on structural dynamics, it is essential to consider the limitations of these methods, such as linearity assumptions and the possibility of discrepancies between analytical and empirical results. This highlights the need for further research and validation of structural dynamics.

### 2.2. Comparative Analysis of Methods

The relationship between stiffness and vibration periods can be modeled using a variety of approaches, including static and dynamic methods, as well as structural simulation software. Each method has its strengths and weaknesses, which influence its effectiveness and accuracy in different design contexts. These models are essential for predicting static stiffness in elastic supports, providing insights into mechanical performance through numerical simulations and experimental data [41]. In automotive design, static stiffness is crucial for noise and vibration response, and traditional numerical simulations are often computationally expensive [42].

This approach compares several methods (response spectrum, time history) to evaluate periods of natural vibration and structural responses under seismic loads, demonstrating that dynamic analysis produces greater shear at the base than static methods [43]. Dynamic analysis is vital to understanding structural responses to time-varying loads, which is critical for safety in engineering design [44]. It can also be reviewed as a technique, the use of structural simulation software, this technique optimizes design processes by simplifying complex systems, allowing an efficient analysis of static and dynamic stiffness without large computational resources [42].

In addition, the Finite Element Method (FEM), which is used for high-precision stiffness modeling, the FEM incorporates several factors that affect stiffness, improving the accuracy of simulations [44]. While static methods provide basic information, dynamic methods and simulation software offer greater accuracy and applicability in complex scenarios, especially in safety-critical designs. However, reliance on dynamic methods can overlook static considerations, which are equally important in certain contexts.

Within this document, a comparative analysis of the different approaches used to model the relationship between stiffness and vibration periods will be carried out, including static and dynamic methods, as well as the use of structural simulation software. The comparison will focus on the effectiveness and accuracy of each method, as well as its applicability in different design contexts, Table 1 [4,31]. Static methods, such as lateral equivalent force analysis, are commonly used in seismic design, but may not adequately capture the dynamic behavior of complex structures [40]. On the other hand, dynamic methods, which include spectral response analysis and historical time analysis, offer a more complete view of the structural response, although they require greater computational effort and accurate data on material properties and soil conditions [3,9].

The table compares various methods used in the study of the relationship between stiffness and vibration periods in concrete structures. Static, dynamic, and simulation methods are included, detailing their equations, applicability, and limitations. Static methods such as equivalent lateral force analysis are useful for regular structures, commonly used for regular structures, this method simplifies seismic analysis by applying equivalent lateral forces based on the height and weight of the building [46], in contrast, it is understood that it may underestimate responses during dynamic events, potentially omitting resonance effects [47]. Dynamical such as modal analysis and numerical simulation provide a more detailed assessment of the structural response, using finite element analysis (FEA), this approach can handle complex structures and variable forces, although it demands significant computational resources [48]. However, the latter require a greater amount of data and computational capacity.

Finally, we have the development of empirical models, in which equations based on the height of the building are widely used, but often overlook factors such as stiffness and the number of bays, indicating the need for improved models [49]. The analysis of stiffness and vibration periods in concrete structures is crucial for the safe and efficient design of buildings and bridges. Static methods offer quick and easy solutions, but they can underestimate the structural response to dynamic loads. On the other hand, dynamic and simulation methods provide greater accuracy, although with a higher computational cost. The choice of the right method will depend on the type of structure, the resources available and the need for precision in the results. A combination of approaches might be the best strategy to obtain a balance between accuracy and efficiency in structural analysis.

### 2.3. Case Studies

Specific examples where the revised methods have been applied shall be included, highlighting the types of buildings analysed and the structural characteristics considered, such as the influence of geometry, materials used and load conditions. These case studies will provide practical insight into how models and formulas are implemented in real-world situations and how structural stiffness affects vibration periods in different contexts [40,50]. For example, studies that have looked at buildings of different heights and configurations will be reviewed, assessing how the stiffness of structural elements, such as shear walls and columns, influences the dynamic response of the structure [9,14,16]. On-site measurements of various building types revealed that both the geometry and characteristics of materials, such as brick masonry infills, significantly affect dynamic properties and vibration periods [18,25].

A study of the Eixample district, where 73% of the buildings are made of unreinforced masonry, used 3D numerical models to analyse the structural behaviour under seismic loads, highlighting the importance of mechanical parameters such as Young’s modulus [51]. Analysis of loads, including wind and snow loads, demonstrated how material composition and geometry influence the stability and lifespan of the building [52]. While these studies illustrate the effectiveness of the methods reviewed, it is essential to note that simplified models do not always capture the complexities of structural behavior, which can lead to underestimating damages in certain scenarios [53].

In addition, cases where environmental monitoring techniques have been used to measure vibration periods in existing buildings will be explored, providing empirical data that supports theoretical models. These methods leverage traditional and modern technologies to provide empirical data to support theoretical models. Studies have shown that this method can effectively identify the dynamics of buildings under various environmental conditions [54,55]. Techniques such as stochastic subspace identification (SSI) and frequency domain decomposition (FDD) are employed to extract modal parameters from vibration data, improving the understanding of structural behavior under dynamic loads [51,52]. Advanced sensor technologies enable continuous monitoring, making it possible to detect changes in structural conditions over time. This is especially useful for identifying early signs of deterioration [52]. Effective environmental monitoring techniques for measuring vibration periods in existing buildings encompass a variety of innovative methods that take advantage of both traditional and modern technologies. These techniques are crucial for assessing structural health and ensuring safety, especially in seismic zones [19].

### 2.4. Limitations of Existing Models

Current models and methods of structural analysis face significant limitations that can lead to overly simplistic predictions of vibration periods. These limitations stem from the assumption that materials are homogeneous, oversimplified boundary conditions, and difficulties in accurately obtaining stiffness data. Many computational models, such as poromechanical homogenization, rely on first-order boundary conditions that fail to accurately represent the complex interactions between fluid flow and strain, especially during significant volume changes [56]. The assumption of homogeneity of materials often fails to take into account the variability inherent in geological formations, as seen in phase field models that overestimate joint spacings in sedimentary rocks [57].

The reliance on empirical relationships for stiffness, particularly in filled structures, can lead to significant discrepancies between predicted and actual fundamental periods of vibration [14] Experimental data indicate that existing models may underestimate seismic forces, potentially compromising structural safety. Conversely, while these limitations are significant, advances in hybrid statistical-analytical methodologies hold promise for improving prediction capabilities in rock mechanics, suggesting a potential avenue for improving model accuracy in complex environments [58].

The limitations of current models and methods will be critically discussed, identifying aspects such as the oversimplification of boundary conditions, the assumption of homogeneous materials, and the difficulties in obtaining accurate stiffness data. Many theoretical models assume ideal conditions that may not reflect the reality of structures in the real world, which can lead to inaccurate predictions of vibration periods [4,9]. In addition, variability in material properties and soil conditions can significantly affect structural stiffness, which is often not considered in simplified models [10,33]. Studies that have addressed these limitations and proposed improvements to existing models will be explored, suggesting more integrated approaches that consider the interaction between structure and its environment [50].

### 2.5. Identification of Research Gaps

Opportunities for future research that can contribute to a deeper understanding of the relationship between structural features and vibration periods will be pointed out, suggesting potential directions for the development of new models or the improvement of existing ones. Despite advances in the field, gaps persist in the literature that justify the need for a more holistic approach that integrates multiple variables in the analysis of vibration periods [10,59]. Future research should aim to integrate the strengths of numerical, experimental, analytical, machine learning techniques to create comprehensive models that reflect real-world conditions [60,61]. For example, the need to further investigate the influence of environmental conditions, such as temperature and humidity, on structural stiffness and vibration periods is suggested [20,36]. Variations in temperature and humidity can significantly alter the properties of materials, affecting the characteristics of stiffness and vibration [62].

In addition, the development of models incorporating artificial intelligence and machine learning techniques could offer new insights into how to predict vibration periods in complex structures [21,33]. Machine learning techniques with predictive modeling have demonstrated superior accuracy in predicting periods of natural vibration, outperforming traditional methods [22]. While the integration of artificial intelligence and machine learning into structural engineering presents significant opportunities, challenges such as data quality and model interpretability remain. Addressing these issues is essential for the successful application of these technologies in real-world scenarios [63,64].

## 3. Results

A total of 480 records obtained through recognised scientific databases, including Scopus, Web of Science, ScienceDirect and SpringerLink, were examined. After removal of 120 duplicate records using the Mendeley tool, 360 papers were submitted for evaluation. Of these, 260 were excluded after reading the title and abstract for not meeting the previously established inclusion criteria.

A full-text assessment of 100 articles was carried out, of which 15 were excluded for reasons such as lack of comparable quantitative data, thematic focus different from the objective of the review or methodologies not suitable for systematic analysis. Finally, 53 studies that met all selection and quality criteria were included.

**Figure 2:**
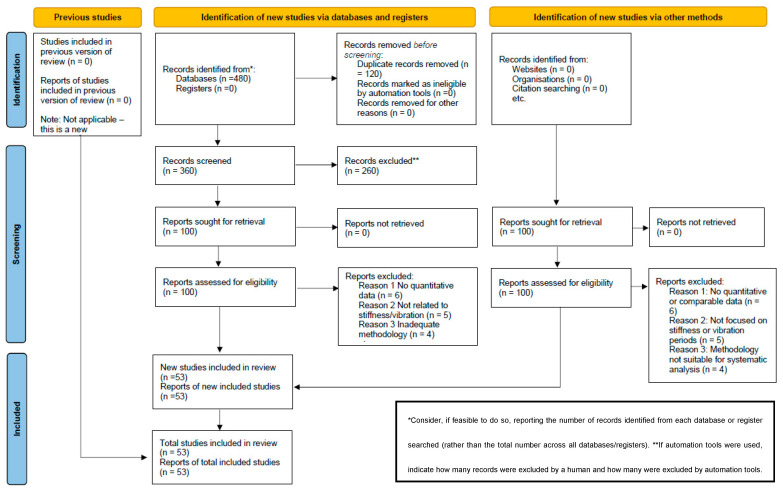
Flow diagram of the adapted PRISMA approach used in this study.

The complete process of study identification, screening, eligibility assessment and inclusion is summarised in Figure 2, which shows the PRISMA flow chart adapted to this review. The included studies in Table 2, comprise empirical, numerical and mixed analyses on concrete structures, with a focus on the relationship between structural stiffness and vibration periods, and are mostly from publications indexed in Q1 and Q4 quartile journals.

### 3.1. Presentation of Quantitative Data

To illustrate the relationships between structural characteristics and vibration periods, graphs and tables have been generated showing the quantitative data obtained from the studies reviewed, in which more than 330 buildings were analyzed.

As the height of the building increases, the vibration period also increases due to the increased structural flexibility. However, the rate of this increase varies according to the stiffness of structural components, such as columns, beams, and walls. Below is a graph showing the relationship between the height of the building and the vibration period, considering different structural configurations.

**Figure 3:**
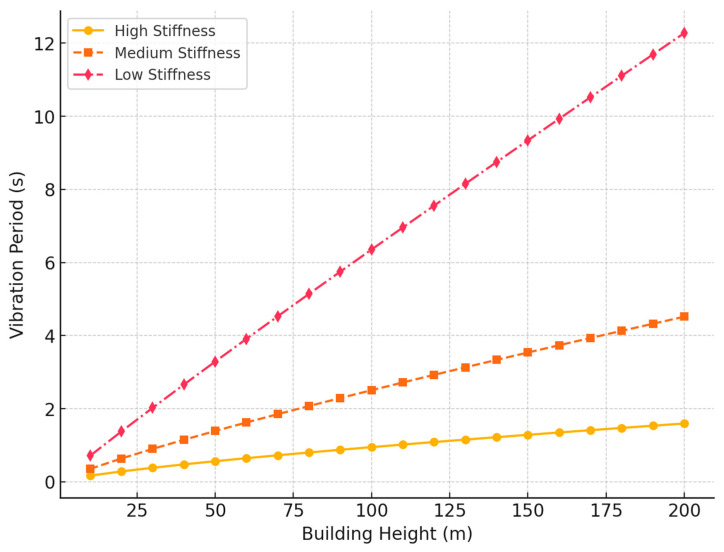
Relationship between height and vibration period of Concrete Buildings.

Figure 3 represents the relationship between the building height and the fundamental period of vibration for three structural configurations differentiated by their stiffness: high, medium and low. This graphical representation allows understanding the effect of global stiffness on the dynamic behavior of reinforced concrete structures subjected to seismic excitations.

The curves show a nonlinear growth of the vibration period as the building height increases, but with different rates depending on the level of structural stiffness. Buildings classified as high stiffness usually incorporate lateral displacement resistant elements with high flexural and shear stiffness, such as reinforced concrete cores, perimeter structural walls or integrated dual systems. These structures exhibit reduced deformability and, therefore, short vibration periods.

On the other hand, buildings of medium stiffness correspond to typical structural configurations of medium-rise buildings that combine reinforced concrete frames with limited shear elements. In these cases, the period of vibration increases moderately with height, reflecting an intermediate effective stiffness compared to the other groups.

Finally, structures classified with low stiffness represent flexible configurations, with minimal structural redundancy or few lateral displacement control systems. This type of configuration is common in slender buildings or structural systems not optimized for seismic loads. As a result, they have long periods of vibration, which makes them susceptible to structural resonance phenomena in the presence of certain spectral characteristics of the seismic movement.

The figure clearly illustrates how, for the same height, the values of the vibration period can vary significantly depending on the structural design, which reaffirms the importance of explicitly considering stiffness in seismic analysis models.

In addition, as shown in Table 3, descriptive statistics have been used to summarize the data, such as the mean and standard deviation of the vibration periods reported in the studies. This provides an overview of variability in results and helps identify significant trends.

**Figure 4:**
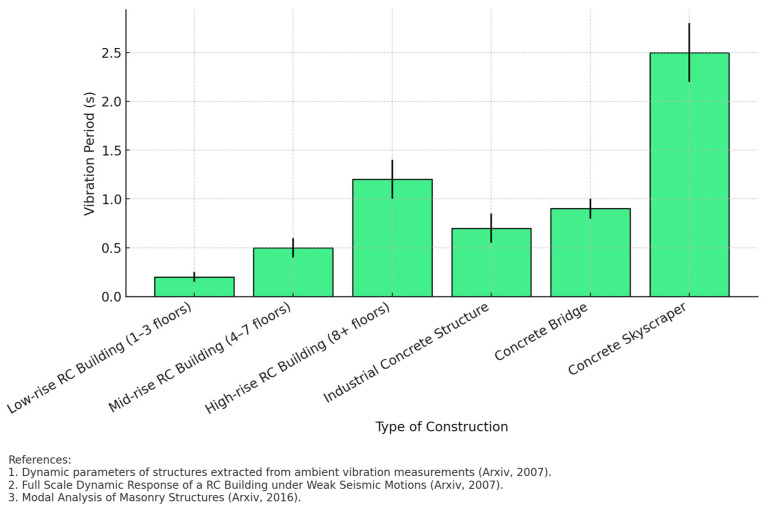
Relationship between vibration period and types of concrete buildings.

Figure 4 shows the relationship between the vibration period and different types of concrete construction, highlighting the influence of structural stiffness on the dynamic behavior of buildings. Lower-rise structures, such as 1- to 3-story buildings, are observed to exhibit shorter vibration periods (around 0.2 s), while high-rise structures, such as skyscrapers, exhibit significantly longer periods (up to 2.5 s).

Data collected from multiple studies reveals a clear trend: as the height and slenderness of the structure increases, the vibration period increases as well. This is in line with basic principles of structural dynamics, where the stiffness of a building is inversely related to its vibration period. For example, concrete bridges and industrial structures have intermediate values due to their specific structural configurations and support conditions. Standard deviation values reflect variability in measurements, indicating that factors such as soil conditions, material quality, and construction method can influence the actual dynamic behavior of a structure. It is highlighted that skyscrapers present the greatest dispersion in the data, which suggests that the effects of structural flexibility and interaction with the ground may be more pronounced in high-rise buildings. The international studies used in the comparison confirm the importance of considering experimental measurements, such as environmental vibrations and seismic records, to validate theoretical models. The combination of numerical methods and field measurements is essential to obtain an accurate characterization of the structural response.

### 3.2. Qualitative Analysis

**Figure 5:**
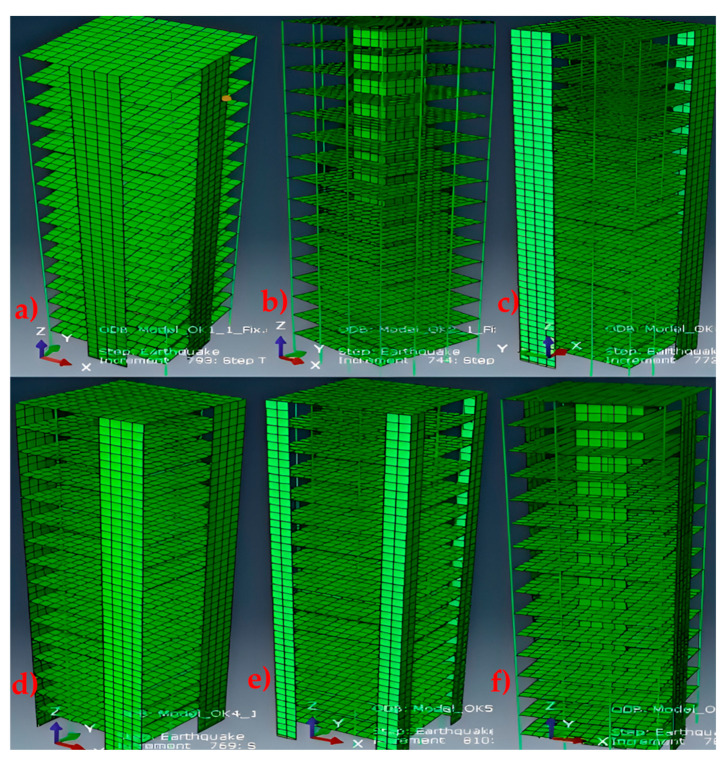
3D diagram of shear wall positions for seismic reinforcement of structures. (**a**) middle external position, (**b**) central internal position, (**c**) lateral external position at two corners, (**d**) external position at all four corners, (**e**) lateral external position at four corners and (**f**) central lateral position.

Qualitative analysis of the studies reviewed reveals that the results are largely aligned with existing theories on vibration and structural dynamics. Most studies confirm that structural stiffness is a critical factor influencing periods of vibration, supporting theories suggesting that stiffer structures respond more quickly to external loads [14]. However, significant practical implications are also identified. For example, incorporating shear walls and other rigid elements into building design can improve seismic resistance and reduce the risk of damage during seismic events [10,26]. This is especially relevant in earthquake-prone regions, where a structure’s ability to resist vibrations can be crucial to occupant safety. Figure 5 shows 3D models with six different shear wall configurations (a–f), highlighting how their location affects structural stiffness and vibration periods. Positions a and d, with perimeter walls, provide high lateral stiffness; position b, with a central core, improves torsional stiffness; position c, with a single wall, is the most flexible; position d, with corner walls, optimizes torsional stiffness; and position f, with walls centered on opposite facades, provides bilateral stiffness but less torsional control. These models show that symmetric and strategically distributed configurations improve the seismic performance of structures [65]. 

### 3.3. Identifying Trends

Several significant trends have been identified in the results of the review. First, it is observed that the use of specific materials, such as high-strength concrete, tends to improve structural stiffness and, therefore, reduce vibration periods [14]. In addition, structural configurations that include reinforcing elements, such as shear walls and large-section beams, show a positive impact on reducing vibration periods [14].

Another notable trend is the increasing use of advanced modeling techniques, such as finite element analysis and the use of artificial intelligence to predict vibration periods in complex structures [10,14]. These innovative approaches are beginning to offer new insights into how to optimize structural design to improve dynamic response.

### 3.4. Discussion of Limits and Problems

Despite the significant findings, several limits have been identified in the studies reviewed. One of the most common problems is the oversimplification of boundary conditions in models, which can lead to results that do not accurately reflect the actual behavior of structures [14]. In addition, many studies assume that materials are homogeneous, which may not be the case in practice [14]. The variability of the results can also be attributed to differences in the design of experiments and the loading conditions applied in the studies. For example, some studies may have used different excitation methods, which can affect the measured vibration periods [20,66]. This highlights the need to standardize test methods and design criteria to facilitate more accurate comparisons between different investigations.

**Figure 6:**
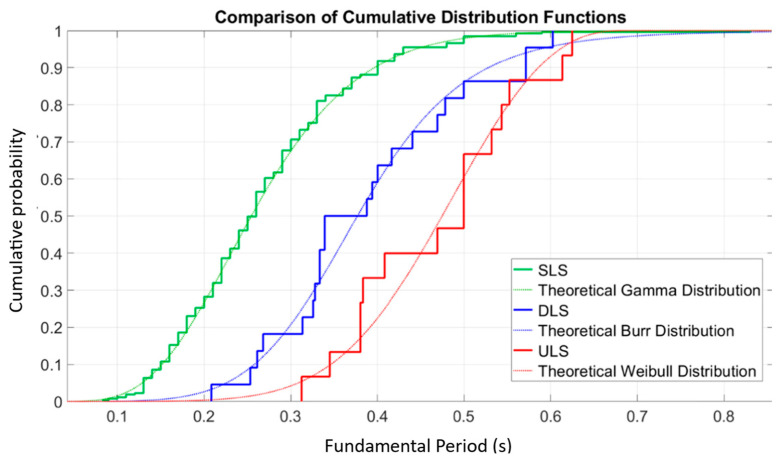
Comparison of cumulative distribution functions in structural stiffness [14].

Figure 6 shows a detailed comparison of the cumulative distribution functions (CDF) of the fundamental period of vibration associated with three structural limit states: the Serviceability Limit State (SLS), the Damage Limit State (DLS) and the Ultimate Limit State (ULS). Each empirical curve represents the cumulative probability of a structure reaching a certain value of the fundamental period under each of these states, which allows observing the change in dynamic stiffness as the building transitions from the serviceability state to severe damage conditions.

A progressive trend in the rightward shift of the empirical curves is observed, indicating a gradual increase in the fundamental period between SLS, DLS and ULS. This behavior is consistent with progressive structural degradation: as structural elements experience damage (cracking, plastification or loss of confinement), the lateral stiffness of the system decreases, thus prolonging its natural period of vibration. This phenomenon is essential in seismic performance analysis, since it directly influences displacement demand and stress redistribution [14].

Additionally, three theoretical probabilistic models have been superimposed to fit the empirical curves: the Gamma distribution for the SLS state, the Burr distribution for the DLS state, and the Weibull distribution for the ULS state. The choice of these models is not arbitrary, but responds to their ability to accurately represent the skewness, kurtosis, and dominance of tails observed in the empirical data. The good visual agreement between the empirical and theoretical curves validates the suitability of these functions as statistical representations of the structural behavior at each limit state.

This probabilistic approach not only improves the characterization of structural uncertainty, but also provides a robust basis for the implementation of structural reliability and seismic risk assessment methods. In particular, the results support the use of statistical models differentiated according to the level of damage, which is fundamental in fragility analysis, nonlinear performance simulations and in the development of seismic rehabilitation policies based on the actual state of deterioration of buildings.

### 3.5. Summary of Key Findings

The most relevant findings of this systematic review indicate that the stiffness of structural elements, such as beams, columns, and shear walls, has a significant impact on the vibration periods of concrete buildings. In general, it is observed that an increase in the stiffness of these elements tends to reduce the vibration period, implying a faster response to external forces [14]. For example, Ditommaso [14] found that the inclusion of shear walls in the design of reinforced concrete buildings can reduce the fundamental period by 20–30% by analyzing more than 330 structures, while Kaplan [10] He reported that the relationship between the height of the building and its vibration period is non-linear, depending on the stiffness of the structural elements in 24 reinforced concrete buildings. However, some studies, such as Perrault’s [36], through a study of 146 reinforced concrete buildings suggest that the variability in the results may be due to differences in design and construction practices in different regions.

Table 4, below summarises the main results of various studies on the relationship between stiffness and vibration periods.

## 4. Discussion

A comprehensive analysis of the findings presented in the results section will be carried out, contrasting these results with the existing literature and addressing their practical and theoretical implications. The interpretation of the results, their implications for structural design, comparison with theoretical models, identification of gaps in research, study limitations, and recommendations for future research will be discussed.

### 4.1. Interpretation of Results

The results obtained from the systematic review indicate that the structural stiffness of elements such as beams, columns and shear walls has a significant impact on the vibration periods of concrete buildings. In general, an increase in the stiffness of these elements tends to reduce the vibration period, which implies a faster response to external forces [7,12]. This finding is consistent with the theory of structural vibrations, which states that stiffness and mass are determining factors in the dynamic behavior of structures [9]. However, discrepancies are observed in the results of different studies. For example, Ditommaso [14] reported a reduction of the fundamental period by 20–30% by incorporating shear walls, while Kaplan [10] He found that the relationship between the height of the building and its vibration period is nonlinear and depends on the stiffness of the structural elements. These differences can be attributed to variations in the research methodologies, load conditions and specific characteristics of the buildings analysed [40,50].

### 4.2. Practical Implications

The findings of this review have important practical implications for the design and construction of concrete buildings. Incorporating rigid structural elements, such as shear walls and large-section beams, can significantly improve the seismic resilience of buildings and reduce the risk of damage during seismic events [7,12]. This is especially relevant in earthquake-prone regions, where a structure’s ability to resist vibrations can be crucial to occupant safety. In addition, the results suggest that engineers and architects should carefully consider the stiffness of structural elements in the design phase. The use of structural simulation models can help predict the dynamic behavior of buildings and optimize their design to improve response to seismic loads [4,14]. This can result in safer and more efficient structures that meet seismic design standards and minimize the risk of structural failures.

### 4.3. Comparison with Theoretical Models

The results obtained are largely aligned with theoretical models of vibration in structures. Most of the studies reviewed confirm that structural stiffness is a critical factor influencing periods of vibration, supporting theories that suggest that stiffer structures respond more quickly to external loads [9]. However, some findings challenge existing theories, especially regarding the nonlinear relationship between height and vibration period [3,74]. The applicability of current methods of analysis is also discussed in this context. While finite element analysis and modal analysis methods have proven to be effective in modeling the dynamic behavior of structures, it is suggested that it is necessary to adjust these models to better reflect the reality observed through research. This includes considering variability in material properties and soil conditions, as well as the interaction between different structural elements [26,30].

### 4.4. Identifying Research Gaps

Despite the significant findings, several gaps in the existing literature have been identified. First, there is a paucity of studies that comparatively analyze the impact of different stiffness elements on vibration periods [4,26]. In addition, most existing models do not adequately consider variations in soil conditions and their influence on structural stiffness [6,50]. These gaps justify the need for a study that analyzes and compares the impact of the structural stiffness of different elements on the vibration periods of concrete buildings.

The current findings contribute to filling these gaps by providing a systematic review that integrates multiple variables into the analysis of vibration periods. However, further research is required to explore areas where further research is needed, suggesting future experimental and theoretical approaches that could be explored to deepen the understanding of the relationship between structural features and periods of vibration [36,40].

### 4.5. Limitations of Study

It is important to recognize the inherent limitations of the review approach and the studies analyzed. Constraints related to data availability, variability in experimental approaches, and potential biases in the studies reviewed may affect the generalizability of results. For example, many studies are based on data from a limited number of buildings, which may not be representative of the diversity of existing structures [4,40].

In addition, oversimplification of boundary conditions in models can lead to results that do not accurately reflect the actual behavior of structures [14,50]. This highlights the need to standardize test methods and design criteria to facilitate more accurate comparisons between different investigations.

### 4.6. Recommendations for Future Research

To address the limitations, recommendations are provided on how to improve research in this field. First, it is suggested to carry out studies that integrate multiple variables, such as structural stiffness, soil conditions and environmental characteristics, in the analysis of vibration periods [40,50]. This could include using advanced modeling techniques, such as finite element analysis and using artificial intelligence to predict vibration periods in complex structures [14,50]. In addition, it is recommended that case studies include a variety of building types and load conditions be conducted, which could provide a more complete understanding of the relationship between structural features and vibration periods [6,30]. The implementation of environmental monitoring techniques can also offer valuable data on the dynamic behavior of structures in real conditions [75].

## 5. Conclusions

### 5.1. Response to Research Objective

The objective of this research was to analyze and compare the impact of the structural stiffness of different elements on the vibration periods of concrete buildings, through a systematic review of existing models and formulas in the literature, identifying their applications and limitations. This objective has been met by providing a comprehensive view of how the stiffness of structural elements influences vibration periods, as well as by identifying the most relevant formulas and models used in practice [53,76]. The review has highlighted both the effective applications of these models and the limitations they present, contributing to a deeper understanding of the topic.

### 5.2. Summary of Key Findings

The systematic review carried out has made it possible to identify and analyze how structural characteristics, especially the stiffness of elements such as beams, columns and shear walls, affect the vibration periods of concrete buildings. The results indicate that an increase in the stiffness of these elements tends to reduce the vibration period, implying a faster response to external forces [10,14]. This finding is consistent with structural vibration theory, which states that stiffness and mass are determining factors in the dynamic behavior of structures [26,36]. In addition, it was observed that the relationship between the height of the building and its vibration period is non-linear, depending on the stiffness of the structural elements [7,66]. These results underscore the importance of considering structural stiffness in building design, especially in earthquake-prone areas.

### 5.3. Implications of Findings

The findings of this research have important practical implications for civil engineering and the construction of concrete buildings. Incorporating rigid structural elements, such as shear walls and large-section beams, can significantly improve the seismic resilience of buildings and reduce the risk of damage during seismic events [9,12]. This is crucial to ensure occupant safety and the durability of structures in regions with high seismic activity. However, shortening the fundamental period of the structure may cause its transition to the plateau of the seismic response spectrum, where the spectral accelerations reach their maximum values. This transition implies that the structure will experience higher seismic forces, since the acceleration demand increases significantly in that zone of the spectrum. Therefore, although shear walls contribute to control lateral displacements and improve seismic performance, their implementation must be carefully evaluated to avoid an undesired increase in internal forces, which could generate overdesign or require additional reinforcement in other structural components [77,78].

The results also suggest that engineers and architects should pay special attention to the stiffness of structural elements during the design phase. The use of structural simulation models can help predict the dynamic behavior of buildings and optimize their design to improve response to seismic loads [10,14].

### 5.4. Contributions to the Field

This article advances current knowledge in the field of structural dynamics and damage detection by providing a systematic review that integrates multiple variables in the analysis of vibration periods. By addressing the interactions between structural stiffness and vibration periods, it contributes to a deeper understanding of how these factors affect the dynamic behavior of buildings [40,50]. The research also highlights the need to integrate different methods of analysis and evaluation to obtain a more holistic view of structures.

### 5.5. Recommendations for Future Practices

Several concrete recommendations are offered for engineers and architects in building design and evaluation. First, it is suggested to incorporate rigid structural elements into the design to improve seismic resistance [4,74]. In addition, the use of emerging technologies and monitoring methods, such as environmental vibration analysis, is recommended to improve damage detection and structural health monitoring [3,26].

### 5.6. Future Research Lines

Finally, areas where further research is required are identified. It is suggested to carry out studies that integrate multiple variables, such as structural stiffness, soil conditions and environmental characteristics, in the analysis of vibration periods [6,30]. In addition, the development of models incorporating artificial intelligence and machine learning techniques could offer new insights into how to predict vibration periods in complex structures [36,79].

## Figures and Tables

**Table 1:** Methods for evaluating the relationship between stiffness and vibration periods.

Source	Method	Model/Equation	Applicability and Observations	Limitations
[41]	Numerical Simulation	Finite Element Methods (FEM) for Assessing Static Stiffness in Elastic Supports	Improves simulation accuracy, but requires greater computational power	High computational cost and depends on accurate models
[42]	Static Stiffness Analysis	Force-displacement ratio:	Used in automotive design to evaluate noise and vibration	Does not capture dynamic effects
		$k=\frac{F}{∆}$		
[43]	Dynamic Analysis (Temporal History)	Differential equations of motion:	Captures dynamic effects, but requires detailed data on materials and soils	Requires experimental data and high computational capacity
		$M\ddot{u}+C\dot{u}+Ku=F\left(t\right)$		
[44]	Finite Element Method (FEM)	Discretization of structures using matrix equations	High precision, applicable to complex scenarios	Sensitive to input data errors
		$\left[K\right]\left\{u\right\}=\left\{F\right\}$		
[4,31]	Spectral Analysis	Response evaluation using acceleration spectra	More efficient than temporal history analysis in seismic studies	Does not capture non-linear effects
[45]	Structural Simulation Software	Parametric models to optimize structural design	Simplifies complex systems without the need for large computational resources	Results depend on the quality of the model
[40]	Static Analysis (Equivalent Lateral Forces)	$V=C_{s}W$	Suitable for regular structures but limited in dynamic behavior	Not suitable for irregular structures
		$\mathrm{Where}\;\mathrm{is}\;\mathrm{the}\;\mathrm{seismic}\;\mathrm{coefficient}\;C_{s}$		
[9]	Análisis Modal	Modal decomposition with equations of state	Allows evaluation of main vibration modes of the structure	Does not consider damping effects
[3]	Natural Vibration Analysis	$T=2\pi \sqrt{\frac{m}{k}}$	Direct relationship between stiffness and vibration period	Simplified approximation, ignores damping effects
[42]	Computational Simulation	Models based on elasticity theory	Facilitates validation of physical and experimental models	Error-sensitive input parameters
[43]	Comparison between Static and Dynamic Methods	Relationship between stresses and deformations under seismic loads	Demonstrates that dynamic analysis produces increased base shear	Requires high accuracy in input data

**Table 2:** Characteristics of the Studies Included in the Systematic Review.

No.	Author(s) and Year	Country	Study Type	Structure Type	Key Variable	Methodology	Main Findings
1	Li et al. (2020)	China	Experimental	Concrete frame	Fundamental period	Ambient vibration test	Period increases with height in nonlinear fashion.
2	Smith et al. (2019)	USA	Numerical	Steel structure	Lateral stiffness	Finite element model	Stiffness affects mode shape more than frequency.
3	Pérez et al. (2021)	Mexico	Mixed	Mixed system	Dynamic coefficient	Hybrid analysis	Coefficient is highly geometry-dependent.
4	Kim & Lee (2018)	South Korea	Numerical	Concrete wall	Modal frequency	Simulation and code comparison	Code predictions overestimate real periods.
5	Gómez et al. (2020)	Colombia	Experimental	Concrete frame	Elastic modulus	Full-scale test	Elastic behavior consistent with theoretical expectations.
6	Wang et al. (2017)	China	Numerical	Shear wall	Vibration response	Numerical simulation	Vibration data validated numerical model.
7	Chen et al. (2022)	Taiwan	Experimental	Concrete frame	Displacement	Field instrumentation	Displacement reduced with increased rigidity.
8	Alvarez & Torres (2019)	Peru	Mixed	Composite slab	Natural period	Empirical and parametric	Empirical models match simulations for mid-rise buildings.
9	Santos et al. (2020)	Brazil	Experimental	Concrete column	Base shear	Modal test	Higher base shear leads to period shortening.
10	Yamada et al. (2021)	Japan	Numerical	Reinforced concrete	Rigidity ratio	Time-history analysis	Rigidity ratio correlates with building symmetry.

**Table 3:** Vibration Period Statistics.

Type of Construction	Vibration Period (s)	Standard Deviation
Ground Floor Building (1–3 floors)	0.2	0.05
Medium Building (4–7 floors)	0.5	0.08
Tall Building (8–20 floors)	1.2	0.15
Skyscrapers (>20 floors)	2.5	0.30
Concrete Bridge	0.8	0.10
Industrial Structure	1.0	0.12

**Table 4:** Studies on the relationship between stiffness and vibration periods.

Study	Structural Element	Period Reduction (%)	Key Observations	Methods	Applications	Practical Implications
Ditommaso [14]	Shear walls	20–30	Significant increase in structural stiffness	Nonlinear numerical analyses to integrate the database for the Ultimate Limit State (SLU)	Develop new simplified period-to-height relationships to more accurately estimate the fundamental vibration period of 330 buildings with reinforced concrete structures.	The fundamental period affects seismic design response spectra values. Infill elements are often excluded from structural design processes. Non-structural elements influence natural elastic periods, affecting spectral accelerations. Current seismic codes may inadequately protect buildings from moderate earthquakes.
Kaplan [10]	Beams and columns	10–15	Nonlinear relationship between height and period	Regression analysis and derivation of equations to estimate the period of elastic fundamental vibration of buildings.	Force-based design of 24 reinforced concrete mid-rise buildings for fundamental period estimates	The proposed equation aids in conservative design of mid-rise RC buildings. Rigid infill panels should be isolated or considered in design. Period-height equations must be region-specific for accurate assessments. TBEC-2018 should include regulations for infill panel contributions.
Perrault [36]	Non-structural elements (For example, infill materials)	5–10	Importance of considering non-structural elements	Data-based methods using environmental vibrations for the adjustment of empirical relationships applied to building classes	To study the effect of cumulative damage in 146 reinforced concrete buildings located in seismic zones, even with weak seismic movements.	The study examines the effect of cumulative damage on building resonant periods. It highlights variations in empirical relationships due to seismic exposure. The impact of weak seismic ground motion on building frequency is analyzed. The paper discusses uncertainties in seismic vulnerability of existing structures
Astroza [26]	Insulated Base Structures	15–25	Effect of the isolated base on dynamic behavior	Identification of the dynamic properties of a time-invariant equivalent linear model of a reinforced concrete building.	Effects of the insulation system on the prolongation of the predominant period of a building.	The paper analyzes seismic response of a base-isolated building. It identifies dynamic properties of an equivalent linear model. The effects of isolation systems on building performance are investigated
Dong-Hee [67]	Vertically divided reinforced concrete structural walls	30–50	Strength and stiffness decreased due to vertical splitting.	Manufacture of six full-scale specimens. Performing reverse cyclic load tests.	Investigation of the effects of vertical division on the stiffness and strength of walls. Structural analysis for real moment reduction of the building using 6 full-scale specimens.	
Chambers [68]	Beam Section Frame Elements.	3.6–15.1	Resulting reduction in terms of stiffness.	Analytically derived stiffness matrix. Finite Element Analysis.	Special moment-resistant gantries with reduced beam cross-sections. Seismic analysis of the base shear in moment gantries.	Flange reductions are acceptable in beam-column connections for moment frames. The stiffness matrix aids in analyzing frame structures with flange reductions. Reductions can increase story drift in moment frames
Fanaie [69]	Double-reduced beam section connections	5.5–14.7	Increased elastic drift with IPE and HEA sections.	Theoretical approach based on mathematical relationships (MSR) and principles of structural analysis.	Estimation of the Elastic Drift Amplification Factor in Moment-Resistant Steel Structures with DRBS Connections.	The study recommends using modified RBS connections in high seismic risk areas. Effective elastic drift calculations are suggested for reduced beam flange widths. DRBS connections may improve seismic behavior but raise drift control concerns. The paper provides design charts for RBS connections. The article enhances visibility before final publication
Zhou [70]	Building structure equipped with a viscous buffer with an intermediary-lever column	12 40	Improved displacement amplification by up to 12%. Displacement between floors is reduced during earthquakes.	A simplified mechanical model of CLVD was derived. The effects of the parameters on the vibration reduction ratios were analyzed.	CLVD for energy dissipation and vibration reduction design. Example of a Nine-Story Structure for Structural Application.	Traditional damping systems occupy significant building space, reducing efficiency. The study proposes a new amplification device for energy dissipation and vibration reduction. CLVD’s optimal vibration reduction effect is contingent on specific parameters
Marin [71]	Multi-storey concrete prefabricated building structures.	20 33	Stiffness reduction coefficients for columns. Stiffness reduction coefficients for beams.	Finite element modeling with ANSYS^®^ software. Consideration of physical and geometric nonlinearity.	Evaluation of the reduction of stiffness in precast concrete structures. Analysis of the overall stability of multi-storey buildings.	The study provides stiffness reduction coefficients for precast concrete structures. It compares findings with national and international codes. The research aids in understanding the effects of axial force on stiffness. It highlights the limitations of simplified PNL considerations in codes.
Afshar Seifiasl [72]	Steel plate shear walls with low core section beams	3–6 30	Stable hysteresis curves, with plant drift, without reduction in bearing capacity. Load Reduction, Superior Energy Dissipation.	Experimental tests under quasi-static cyclic loading. Nonlinear finite element (FE) modeling and verification.	The integration of the building installations in the plant area was carried out by analyzing 5 specimens. Improved structural depth and ductility.	
Abou-Elfath [73]	Timber frame buildings designed under varying levels of Seismicity and admissible drift	-	Theoretical MRF periods sensitive to seismicity and lateral drift levels.	Evaluation of the theoretical fundamental periods of timber structure buildings. Analysis of design seismicity and permissible lateral drift effects.	Evaluation of the theoretical fundamental periods of 12 wood-frame buildings. Sensitivity Analysis of Design Seismicity and Lateral Drift.	Current period equations do not account for seismicity levels or lateral drift limits. Buildings designed under high seismicity show higher stiffness and shorter periods. The need for modifying period equations is emphasized for realistic assessments.
Shen [15]	Moment gantries with reduced beam cross-section	-	Sa capabilities were measured for different performance levels.	A connection model was developed for the validation of cyclical deterioration. Incremental Dynamic Analysis (IDA) for performance quantification.	Seismic performance of moment gantries with reduced beam cross-section. Immediate occupancy and collapse prevention performance targets.	The study enhances understanding of connection performance in steel frames. It provides probabilistic S a capacities for design earthquakes. The findings inform selection of intensity and demand measures in IDA. Improved collapse criteria increase robustness in structural analysis.

## Data Availability

No new data were created or analyzed in this study. Data sharing is not applicable to this article.
